# Supplementary figures and images for: Hydroxyl carlactone derivatives are predominant strigolactones in Arabidopsis
Source: Plant Direct. 2020 May 8;4(5):e00219. doi: 10.1002/pld3.219 (PMC7207163; doi:10.1002/pld3.219)

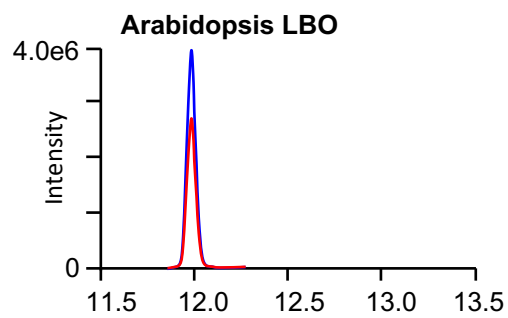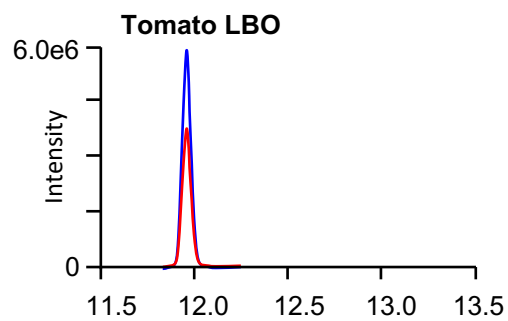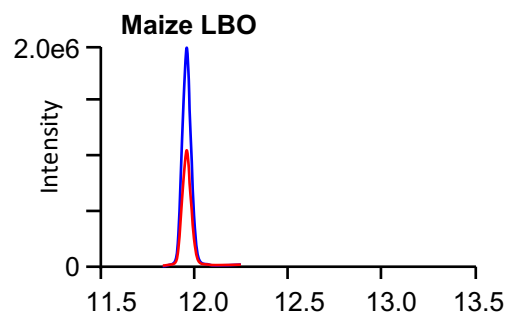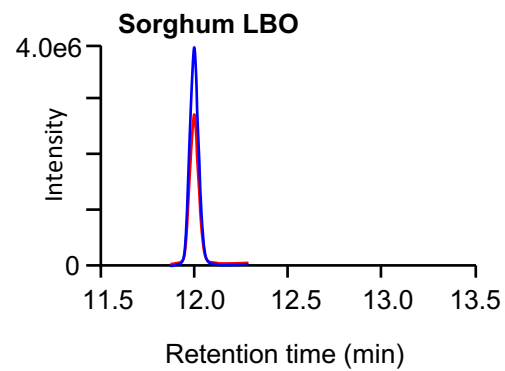

Figure S1

Supplement: Supplementary file 1 — Fig S1 [file PLD3-4-e00219-s001.pdf]

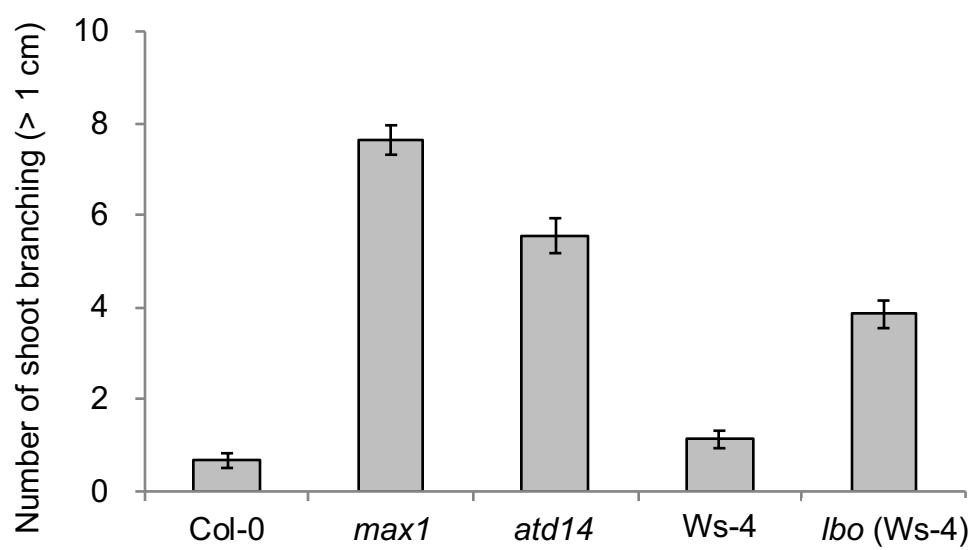

Figure S2

Supplement: Supplementary file 2 — Fig S2 [file PLD3-4-e00219-s002.pdf]

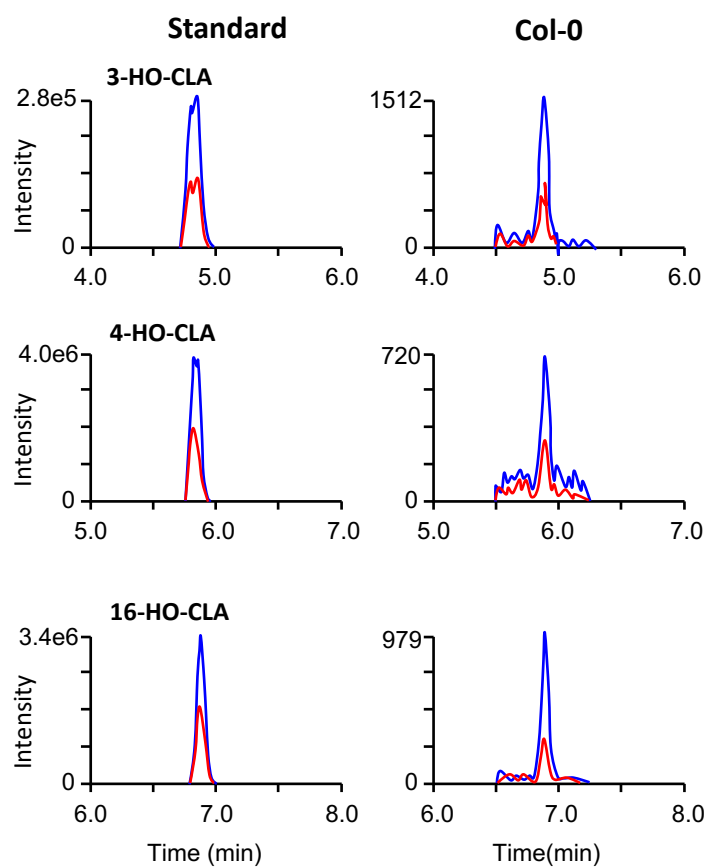

Figure S3

Supplement: Supplementary file 3 — Fig S3 [file PLD3-4-e00219-s003.pdf]
