# Supplementary material for: Hydroxyl carlactone derivatives are predominant strigolactones in Arabidopsis
Source: Plant Direct. 2020 May 8;4(5):e00219. doi: 10.1002/pld3.219 (PMC7207163; doi:10.1002/pld3.219)
Supplement: Supplementary file 4 — Fig S4 [file PLD3-4-e00219-s004.pdf]

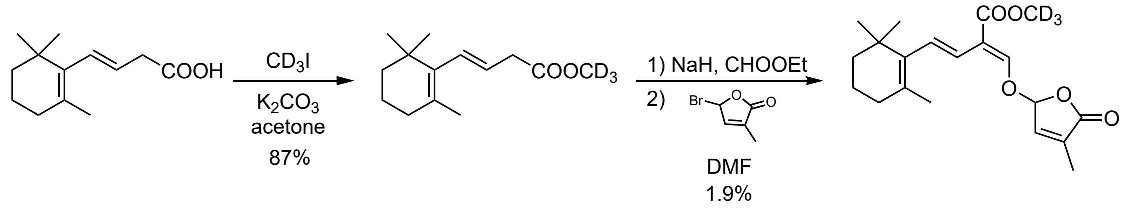

Scheme S1. Synthesis of methyl-d_3 carlactonoate (1'-d_3-MeCLA).

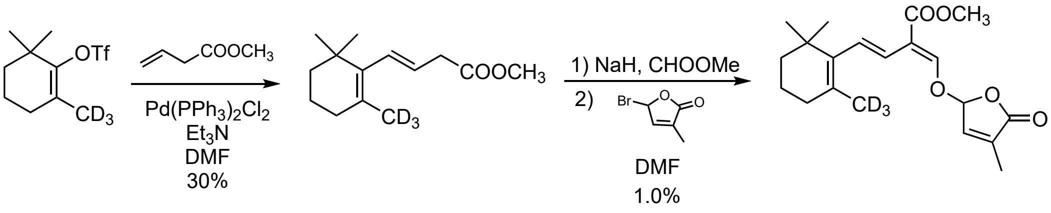

Scheme S2. Synthesis of methyl 18-d_3-carlactonoate (18-d_3-MeCLA).

Figure S4
