# Supplementary material for: Hydroxyl carlactone derivatives are predominant strigolactones in Arabidopsis
Source: Plant Direct. 2020 May 8;4(5):e00219. doi: 10.1002/pld3.219 (PMC7207163; doi:10.1002/pld3.219)
Supplement: Supplementary file 5 — Table S1 [file PLD3-4-e00219-s005.pdf]

Table S1. Primer sequences used for cloning.

Primer name	5' to 3' sequence
LBO-2-F1	AGAACAGTTGTGTTGACCTTC
LBO-2-R1	GGATCTGAAGGTCAACACAAC
LBO-3-F1	GTTATTGATCTCTCTAAGTTCTCTAAAC
LBO-3-R1	TTGTCGGGTTTAGAGAACTTAG
Tomato-LBO-F1	TGTATGCCTAACAAAGCATAGAC
Tomato-LBO-R1	TACATCTCAAGTTAATAGTGTGTCTC
CACC-tomato-LBO-F2	CACCATGGCTCCAGTGCCAAGTTTTC
Tomato-LBO-R2	TTACTTGTCATAGATTTTGGCAAAC
Maize-LBO-F1	GGACGGCTATGAGAAATCTTG
Maize-LBO-R1	GTATTCTATGCAGCTAGACAGTAG
CACC-maize-LBO-F2	CACCATGGCCACCTCACATAAAGAC
Maize-LBO-R2	CTAGGTAGAGGTTTCCTCGGG
Sorghum-LBO-F1	ATGCGATCAAGATTTTGTTGCATTG
Sorghum-LBO-R1	CCAAATGCGACTACGTGGAG
CACC-sorghum-LBO-F2	CACCATGCGATCAAGATTTTGTTGCATTG
Sorghum-LBO-R2	GTTTCCTCACGAGTCAGGAG
