# Supplementary material for: Hydroxyl carlactone derivatives are predominant strigolactones in Arabidopsis
Source: Plant Direct. 2020 May 8;4(5):e00219. doi: 10.1002/pld3.219 (PMC7207163; doi:10.1002/pld3.219)
Supplement: Supplementary file 6 — Supplementary Material [file PLD3-4-e00219-s006.pdf]

Response to reviews of the Plant Journal

Reviewer: 1

Reviewer Report for the Authors

The manuscript “Hydroxyl carlactone derivatives are predominant strigolactones in Arabidopsis” by Kaori et al. describes the identification of the product(s) formed by the enzyme Lateral Branching Oxidoreductase (LBO) that catalyzes a later step in strigolactone biosynthesis. Although, Brewer et al., has already shown that the Arabidopsis LBO acts on methyl calctonoate (MeCLA) and produces a MeCLA + 16 Da product, indicating a hydroxylation reaction, the structure of the product or the position of the hydroxylation have remained elusive. The authors of this manuscript have been able to identify the position of the hydroxylation - by using labeled MeCLA - and claim that LBO converts MeCLA into 1’-HO-MeCLA. Instability and low amount of the product made it impossible to confirm the structure by NMR. Indeed, the identification of this compound as a product of LBO is an achievement! The authors provided further evidence to support their claim, by testing LBOs from other plants and by identifying strigolactone intermediates in Arbidopsis lbo and strigolactone receptor, d14, mutants. The analysis of these mutants revealed the presence of several hydroxylated carlactone and MeCLA species in both mutants, while 1’-HO-MeCLA was only detected in the d14 mutant. By analyzing the OH-MeCLA presence in max1 mutant, they also show that MAX1 is converting hydroxylated carlactones into corresponding acids. These findings are very important, as they may fill a gap in strigolactone biosynthesis and may lead to the identification of the final product of this pathway in Arabidopsis.

Major points:

1- As stated by the authors, the enzyme acts rather as a “demythelase” that converts MeCLA back into CLA. The ratio of CLA to 1'-HO-MeCLA was 100: 1. It is unlikely that LBO catalyzes a reverse reaction of the yet unidentified methyl transferase in planta. However, at the same time, the very low ratio of 1'-HO-MeCLA, compared to CLA, makes it difficult to believe that LBO biological function is the production of this compound. It seems more likely that the authentic LBO substrate is not MeCLA but a different strigolactone intermediate. Alternatively, one could speculate about a protein/enzyme partner that pushes LBO catalysis towards hydroxylation instead of demethylation. In other words, the evidence provided by the authors is not sufficient to claim that 1'-HO-MeCLA is the major product of LBO but indicates that 1'-HO-MeCLA is likely a side product.

We do not claim that 1'-HO-MeCLA is a major product of LBO. However, it is an important fact that 1'-HO-MeCLA is endogenous in Arabidopsis *atd14* mutants and LBO converts MeCLA into 1'-HO-MeCLA. As mentioned in the manuscript, it is possible that 1'-HO-MeCLA is a precursor for the true shoot branching inhibitor in Arabidopsis.

2- The authors excluded that LBO can produce 4-HO- or 16-HO-MeCLA. This claim needs further experimental confirmation.

4-HO-MeCLA and 16-HO-MeCLA accumulated in *lbo* mutant, and therefore we can exclude the possibility that LBO produces these hydroxyl MeCLA. These compounds could be substrates for LBO but we could not find their LBO products so far except for the corresponding HO-CLAs.

3- A quantitative analysis of the different hydroxylated MeCLA and carlactone species is missing and would be very useful. Which hydroxylated form is the major one?

Yes, it is very useful to quantify HO-MeCLAs and related compounds. Unfortunately, CL derivatives including HO-MeCLAs are highly unstable and thus recovery rates from tissue samples are rather low. Therefore isotope-labeled internal standards should be used for quantification of these compounds. At the moment, such a standard is available only for CL. Simple comparison of peak areas of different compounds means nothing.

3- The correlation between 1'-HO-MeCLA formation and shoot branching postulated by the authors is quite speculative. The authors did not provide direct evidence to support the function of 1'-HO-MeCLA in shoot branching, but used lbo-2 and lbo-3 mutants. I recommend to profile all strigolactones in the three different *lbo* mutants and - if feasible – to compare the conversion of MeCLA by lbo-2 and lbo-3 versions of the enzyme with that of wild-type.

We did compare enzymatic activity of LBO-2 and LBO-3 expressed in *E. coli*. As described, LBO-2 was functional but its activity was low. LBO-3 was as active as LBO. We should conduct kinetics test, but, unfortunately we don't have a sufficient amount of standards and isotope-labeled MeCLA to perform kinetics study.

Minor points:

1- Please change [MeCLA+16] Da into [MeCLA+16Da].

Revised as suggested.

2- Authors should show the negative control of the in vitro assay.

Our previous study (Brewer et al. 2016) showed that 1'-HO-MeCLA could not be detected in enzyme assays in the absence of LBO or 2OG.

Reviewer: 2

Reviewer Report for the Authors

Strigolactones (SLs) are important regulator not only for plant development, but also for communicating with soil microbes and parasitic plants, yet its biosynthetic pathway is not completely elucidated. Indeed, the lack of our knowledge about the identity of active SL in Arabidopsis is somewhat surprising. The manuscript by Yoneyama et al describes the identification of 1'-OH-MeCLA as a product of the downstream biosynthetic enzyme called LBO. This was enabled by feeding recombinant LBO protein with candidate substrate called MeCLA which is labelled with deuterium at different positions. The authors confirmed the presence of 1'-OH-MeCLA in Arabidopsis d14 (SL receptor) mutant by LC-MS, but the peak was not seen in loss-of-function mutant in LBO. However, the production of 1'-OH-MeCLA in vitro was rather minor and the major product was CLA, which is believed as a precursor of MeCLA (at the ratio of 1'-OH-MeCLA:CLA = 1:100). Similar trend was seen in LBO ortholog proteins from tomato, maize and sorghum. The authors also tested some variants of hydroxylated MeCLAs, which could be produced by an upstream enzyme called MAX1.

Although the identification 1'-OH-MeCLA as the unknown product of LBO (MeCLA+16Da) is a progress, I feel claiming its role in SL biology is premature. One scenario is that, as the authors expected, 1'-OH-MeCLA (or its downstream product) is the active SL in Arabidopsis, implying that 1'-OH-MeCLA (or its downstream product) binds to AtD14 and activates SL signaling better than MeCLA. The other scenario is that MeCLA is not a genuine substrate of LBO and 1'-OH-MeCLA does nothing to SL signaling. Without providing evidences for the function of 1'-OH-MeCLA, we cannot tell which is true. We are thus still left with the question of what is the identity of active SL in Arabidopsis.

Many thanks for your comments and suggestions.

Due to instability of 1'-OH-MeCLA, we cannot prepare a sufficient amount of this compound to conduct bioassays by organic synthesis or enzyme assays. So, we would like to simply describe characterization of LBO reaction product along with some biological data in this manuscript.

Minor comments:

P7L1; 1'-HO-MeCLA is present in atd14 mutants but not in lbo mutants

The comparison between lbo and atd14 mutant is unfair. To make fair comparison, lbo should be compared with lbo/atd14 double mutant. I am not sure whether the lack of 1'-HO-MeCLA is due to lbo mutation, or even with LBO it is difficult to detect without atd14 mutation.

Unfortunately, we don't have any *lbo/atd14* mutant lines.

P8L1; statements about LBO orthologs in tomato, maize and sorghum

do these plant species produce MeCLA? Whether MeCLA is a natural substrate for these LBO orthologs is an important information for the readers.

So far, MeCLA could be found from Arabidopsis plants but not from these crops. However, we think these crops do produce MeCLA. For example, non-canonical SLs in maize appear to be derived from MeCLA. In tomato and sorghum, MeCLA would be quickly converted into SLs. Accordingly, MeCLA have not yet been detected from these crops.

FigS2;

to claim correlation, the data about lbo-2 and lbo-3 should be presented side by side.

Sorry, we cannot follow this question.
